# Supplementary material for: Probiotic Properties and Safety Evaluation of Lactobacillus plantarum HY7718 with Superior Storage Stability Isolated from Fermented Squid
Source: Microorganisms. 2023 Sep 8;11(9):2254. doi: 10.3390/microorganisms11092254 (PMC10534859; doi:10.3390/microorganisms11092254)
Supplement: Supplementary file 1 [file microorganisms-11-02254-s001.zip › microorganisms-2587451-supplementary.pdf]

Table S1. List of lactic acid bacterial strains isolated from Korean traditional fermented foods

No.	Species	Sources	No.	Species	Sources
#1	*Lactobacillus plantarum*	Makgeolli	#31	*Lactobacillus paracasei*	Makgeolli
#2	*Lactobacillus plantarum*	Makgeolli	#32	*Lactobacillus paracasei*	Makgeolli
#3	*Lactobacillus plantarum*	Makgeolli	#33	*Lactobacillus paracasei*	Makgeolli
#4	*Lactobacillus plantarum*	Cabbage Kimchi	#34	*Lactobacillus paracasei*	Makgeolli
#5	*Lactobacillus plantarum*	Cabbage Kimchi	#35	*Lactobacillus paracasei*	Cabbage Kimchi
#6	*Lactobacillus plantarum*	Cabbage Kimchi	#36	*Lactobacillus paracasei*	Radish Kimchi
#7	*Lactobacillus plantarum*	Cabbage Kimchi	#37	*Lactobacillus paracasei*	Cabbage Kimchi
#8	*Lactobacillus plantarum*	Cabbage Kimchi	#38	*Lactobacillus paracasei*	Cabbage Kimchi
#9	*Lactobacillus plantarum*	Makgeolli	#39	*Lactobacillus paracasei*	Cabbage Kimchi
#10	*Lactobacillus plantarum*	Cabbage Kimchi	#40	*Lactobacillus paracasei*	Makgeolli
#11	*Lactobacillus plantarum*	Makgeolli	#41	*Lactobacillus paracasei*	Makgeolli
#12	*Lactobacillus plantarum*	Makgeolli	#42	*Lactobacillus paracasei*	Makgeolli
#13	*Lactobacillus plantarum*	Makgeolli	#43	*Lactobacillus paracasei*	Makgeolli
#14	*Lactobacillus plantarum*	Makgeolli	#44	*Lactobacillus paracasei*	Makgeolli
#15	*Lactobacillus plantarum*	Dongchimi	#45	*Lactobacillus paracasei*	Makgeolli
#16	*Lactobacillus plantarum*	Fermented fish	#46	*Lactobacillus paracasei*	Makgeolli
#17	*Lactobacillus plantarum*	Gat Kimchi	#47	*Lactobacillus paracasei*	Makgeolli
#18	*Lactobacillus plantarum*	Siraegi	#48	*Lactobacillus paracasei*	Makgeolli
#19	*Lactobacillus plantarum*	Fermented Squid	#49	*Lactobacillus paracasei*	Makgeolli
#20	*Lactobacillus plantarum*	Makgeolli	#50	*Lactobacillus paracasei*	Makgeolli
#21	*Lactobacillus paracasei*	Cabbage Kimchi	#51	*Lactobacillus paracasei*	Makgeolli
#22	*Lactobacillus paracasei*	Makgeolli	#52	*Lactobacillus paracasei*	Cabbage Kimchi
#23	*Lactobacillus paracasei*	Makgeolli	#53	*Lactobacillus paracasei*	Makgeolli
#24	*Lactobacillus paracasei*	Makgeolli	#54	*Lactobacillus paracasei*	Makgeolli
#25	*Lactobacillus paracasei*	Makgeolli	#55	*Lactobacillus paracasei*	Makgeolli
#26	*Lactobacillus paracasei*	Makgeolli	#56	*Lactobacillus paracasei*	Persimmon Vinegar
#27	*Lactobacillus paracasei*	Makgeolli	#57	*Lactobacillus paracasei*	Makgeolli
#28	*Lactobacillus paracasei*	Makgeolli	#58	*Lactobacillus paracasei*	Makgeolli
#29	*Lactobacillus paracasei*	Makgeolli	#59	*Lactobacillus paracasei*	Makgeolli
#30	*Lactobacillus paracasei*	Makgeolli	#60	*Lactobacillus paracasei*	Green Onion Kimchi

Table S2. COG categories of CDSs in *Lactobacillus plantarum* HY7718.

EggNOG	Function	Number
D	Cell cycle control, cell division, chromosome partitioning	31
M	Cell wall/membrane/envelope biogenesis	159
N	Cell motility	3
O	Posttranslational modification, protein turnover, chaperones	65
T	Signal transduction mechanisms	75
U	Intracellular trafficking, secretion, and vesicular transport	23
V	Defense mechanisms	59
W	Extracellular structures	0
Y	Nuclear structure	0
Z	Cytoskeleton	0
A	RNA processing and modification	0
B	Chromatin structure and dynamics	0
J	Translation, ribosomal structure and biogenesis	147
K	Transcription	243
L	Replication, recombination and repair	232
C	Energy production and conversion	104
E	Amino acid transport and metabolism	201
F	Nucleotide transport and metabolism	88
G	Carbohydrate transport and metabolism	235
H	Coenzyme transport and metabolism	63
I	Lipid transport and metabolism	57
P	Inorganic ion transport and metabolism	154
Q	Secondary metabolites biosynthesis, transport and catabolism	22
R	General function prediction only	0
S	Function unknown	782

1. Materials and Methods

1.1 Cell viability Assays

The MTT assay was performed to confirm cell viability of HY7718. Caco-2 cells were seeded at 1.0 × 10^4 cells per well in 96-well plates, then were stabilized for 24 h in MEM culture medium. Cells were treated with various concentration of HY7718 (10^4, 10^5 and 10^6 CFU/well) for 24 h. And then, 0.5 mg/mL 3-(4,5-dimethyl-2-thiazolyl)-2,5-diphenyl-2H-tetrazolium bromide (MTT) solution was added and reacted for 1-4 hours in a CO_2 incubator at 37 °C. After the reaction is over, the medium was removed and 100 μL of Dimethyl sulfoxide (DMSO) was added into per well. The MTT crystals were sufficiently dissolved, absorbance of eluted solutions was measured at 595 nm by BioTek® Synergy HT Microplate reader (Santa Clara, CA, USA).

2. Results

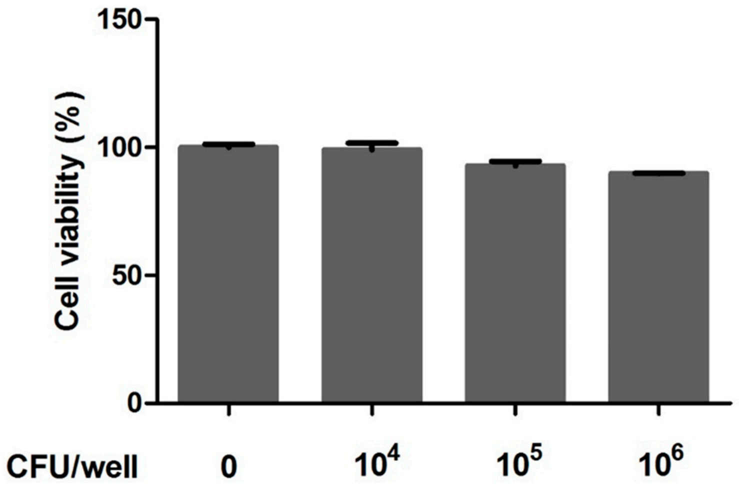

Figure S1. Cell viability of *L. plantarum* HY7718.
